# Oxygen Availability for Porphyrin Biosynthesis Enzymes Determines the Production of Protoporphyrin IX (PpIX) during Hypoxia

**DOI:** 10.1371/journal.pone.0146026

**Published:** 2015-12-30

**Authors:** Shimpei Otsuka, Kentaro Matsumoto, Motowo Nakajima, Tohru Tanaka, Shun-ichiro Ogura

**Affiliations:** 1 Graduate School of Bioscience and Biotechnology, Tokyo Institute of Technology, 4259 B47 Nagatsuta-cho, Midori-ku, Yokohama 226–8501, Japan; 2 SBI pharmaceuticals CO., LTD., Izumi Garden Tower 20F, 1-6-1, Roppongi, Minato-ku, Tokyo, 106–6020, Japan; CINVESTAV-IPN, MEXICO

## Abstract

5-Aminolevulinic acid (ALA), a precursor of porphyrin, is specifically converted to the fluorescent substance protoporphyrin IX (PpIX) in tumors to be used as a prodrug for photodynamic therapy and diagnosis. Hypoxia, a common feature of solid tumors, decreases the efficacy of ALA-based photodynamic therapy and diagnosis. This decrease results from the excretion of porphyrin precursor coproporphyrinogen III (CPgenIII), an intermediate in the biosynthesis of PpIX. However, the mechanism of CPgenIII excretion during hypoxia remains unclear. In this study, we revealed the importance of mitochondrial respiration for the production of PpIX during hypoxia. Porphyrin concentrations were estimated in human gastric cancer cell lines by HPLC. Expression levels of porphyrin biosynthesis genes were measured by qRT-PCR and immunoblotting. Blockage of porphyrin biosynthesis was an oxygen-dependent phenomenon resulting from decreased PpIX production in mitochondria under hypoxic conditions. PpIX production was increased by the inhibition of mitochondrial respiration complexes, which indicates that the enzymes of porphyrin biosynthesis compete with respiration complexes for molecular oxygen. Our results indicate that targeting the respiration complexes is a rationale for enhancing the effect of ALA-mediated treatment and diagnosis.

## Introduction

5-Aminolevulinic acid (ALA) is a precursor in the porphyrin biosynthetic pathway, which produces the bioactive molecule heme. When ALA is administered to cancer patients, cancer cells specifically accumulate the fluorescence precursor protoporphyrin IX (PpIX), although PpIX is converted to heme in normal cells. This specificity is widely used for the photodynamic diagnosis (PDD) of gliomas [1], bladder cancers [2], and prostate cancers [3], allowing their complete resection.

Hypoxia, a pathologic microenvironment that occurs in solid tumors, is caused by their incomplete vascular structure and limited perfusion [4]. Because medicine delivery is difficult in hypoxic regions, hypoxic cancer cells are resistant to chemotherapy [4]. Hypoxic cancer cells also display radioresistance, because molecular oxygen amplifies DNA damage [5,6]. It has also been shown that hypoxia decreases the efficacy of ALA-mediated photodynamic therapy (ALA-PDT) *in vitro* due to a reduction in PpIX accumulation during hypoxia [7,8]. Furthermore, hypoxia inducible factor (HIF), the major regulator of the hypoxic response, promotes the expression of genes associated with angiogenesis, chemoresistance, invasion, and metastasis [9]. Thus, eliminating hypoxic cancer cells is important for the success of treatment.

Heme biosynthesis is altered in hypoxia because the expression levels of various enzymes and transporters involved in heme biosynthesis are modified. The activity of ALA hydrogenase and the expression level of ferrochelatase (FECH), the second and eighth enzymes of porphyrin-heme biosynthesis pathway, respectively, are increased in hypoxia, resulting in an increase in heme biosynthesis [10–12]. On the other hand, the expression levels of hydroxymethylbilane synthase (HMBS) and uroporphyrin synthase, the third and fourth enzymes of porphyrin-heme biosynthesis pathway, respectively, are decreased in hypoxia, resulting in a decrease in porphyrin biosynthesis [10,13]. The expression level of the human ABC transporter ABCG2, previously identified as a PpIX export transporter, is also increased in hypoxia [14]. However, it is unclear whether these changes in expression level affect the ALA-mediated accumulation of PpIX in hypoxia.

Our previous study showed that a precursor of PpIX, coproporphyrinogen III (CPgenIII) is excreted during hypoxia [15]. In addition, the expression level of ABCB6 in the plasma membrane is upregulated during hypoxia, resulting in increased extracellular coproporphyrin III (CPIII) concentrations [15]. However, the mechanism responsible for the blockage of heme biosynthesis at CPgenIII during hypoxia remains unclear. In this study, we revealed the importance of mitochondrial respiration to the production of PpIX during hypoxia. The ability of mitochondria to synthesize PpIX was decreased during hypoxia. This ability was recovered by the inhibition of respiration complexes. These results indicate that targeting mitochondrial respiration is expected to enhance the effect of ALA-PDT in clinical situations.

## Materials and Methods

### Biochemicals

ALA hydrochloride was purchased from Cosmo Oil Co., Ltd. (Tokyo, Japan). Cobalt (II) chloride hexahydrate, cycloheximide, RPMI-1640 medium and antibiotic-antimycotic solution (ABAM) were purchased from Nacalai Tesque (Kyoto, Japan). Deferroxamine mesylate was purchased from Santa Cruz Biotechnology (Dallas, Texas, USA). Dimethyloxaloglycine (DMOG) was purchased from Enzo Life Sciences, Inc. (Farmingdale, New York, USA). Antimycin and Oligomycin were purchased from A.G. Scientific (San Diego, California, USA). Rotenone was purchased from Enzo Life Sciences, Inc. (Farmingdale, New York, USA). Fetal Bovine Serum (FBS) was purchased from Equitech-Bio Inc. (Kerrville, Texas, USA).

### Cell Culture

Human gastric cancer cell lines KatoIII, MKN74, and MKN45 were purchased from the RIKEN Bioresource Center (Tsukuba, Ibaraki, Japan). Human TMK-1 gastric cancer cells were provided by Dr. Tahara (Hiroshima University, Hiroshima, Japan). Cells were maintained under an atmosphere containing 5% CO_2_ at 37°C in RPMI-1640 medium supplemented with 10% (v/v) heat-inactivated FBS and 1× antibiotic–antimycotic mixed stock solution. Cell culture under hypoxic conditions was carried out using AnaeroPack-Kenki 5% (Mitsubishi Gas Chemical Co., Tokyo, Japan).

### Treatment with Pharmacological Inhibitors

CoCl_2_ (100 μM), deferoxamine (100 μM), and dimethyloxalylglycine (1 mM) were used to inhibit prolyl hydroxylases (PHDs) and to activate HIF-1μ. Cycloheximide (10 μg/mL) was used to inhibit protein synthesis. Rotenone (1 μM), antimycin (1 μM), and oligomycin (0.1 μM) were used to inhibit Complexes I, III, and V, respectively. Each inhibitor was added together with ALA for 24 h.

### HPLC Analysis of Porphyrins

Cells (0.5 × 10^6^ cells for KatoIII, MKN74, MKN45 cells and 0.2 × 10^6^ cells for TMK-1 cell) were incubated with 1 mM ALA with or without inhibitors for 24 h under normoxia and hypoxia. Cells were washed twice with phosphate-buffered saline (PBS) and then treated with 0.1 M NaOH. Cellular lysates were prepared by adding 3-fold volumes of dimethyl formamide (DMF)/2-propanol (100:1, v/v) to oxidize coproporphyrinogen III (CPgenIII) to coproporphyrin III (CPIII). These mixtures were then centrifuged to remove proteins, and the supernatants were incubated at room temperature in the dark for 1 day. High-performance liquid chromatography (HPLC) analysis was performed as previously described [15], with some modifications. Briefly, PpIX and CPIII were separated using an HPLC system (Prominence, Shimadzu, Kyoto, Japan) equipped with a reversed-phase C_18_ Column (CAPCELL PAK, C18, SG300, 5 μm, 4.6 mm × 250 mm, Shiseido Co., Ltd., Tokyo, Japan). Elution was started with 100% solvent A and 0% solvent B for 5 min, with a linear gradient of solvent B (0%–100%) for 25 min, and then with solvent B for 10 min. Flow was maintained at a constant rate of 1.0 mL/min, Porphyrins were continuously detected using a fluorospectrometer (excitation, 404 nm; emission, 624 nm). Porphyrin concentrations were estimated using calibration curves constructed using porphyrin standards.

### Quantitative PCR

Total RNA was isolated using the NucleoSpin^®^ RNA II (MACHEREY-NAGEL, Düren, Mannheim, Germany) kit according to the manufacturer’s instructions. Total RNAs (1 μg) were reverse-transcribed to produce first-strand cDNA using the PrimeScript RT reagent Kit with gDNA Eraser (TaKaRa, Shiga, Japan) according to the manufacturer’s protocol (Matsumoto *et al*, 2015). The Thermal Cycler Dice Real Time System (TaKaRa, Shiga, Japan) was used to perform a two-step reverse transcription polymerase chain reaction. The mRNA transcripts were quantified using SYBR Premix ExTaq (TaKaRa, Shiga, Japan). Primers are shown in Table 1.

**Table 1:** Sequences of primers used in real-time PCR.

Gene Symbol	Sense or antisense	Sequence
ALAD	Sense	5’-AACCAGCATAAATACTGCCTGAGGA-3’
	Antisense	5’-TGGCACCTCTAGCAGTCAGGAA-3’
HMBS	Sense	5’-AACAGCCCAAAGATGAGAGTGATTC-3’
	Antisense	5’-AATGTTGCCACCACACTGTCC-3’
UROD	Sense	5’-CCCTGTGCCTTGTATGCATCTG-3’
	Antisense	5’-TGTAGCGATGTGGTCCAAAGTCA-3’
UROS	Sense	5’-CCTGAACAGCTACTATTCCGAGCA-3’
	Antisense	5’-CTTGAGACTGTATGTGAGGCCAGAG-3’
ABCB6	Sense	5’-TTCACTGTGATGCCTGGACAGA-3’
	Antisense	5’-GGATGCAGCCAGAGCTGATG-3’
CPOX	Sense	5’-CACTCCAGGATCCAGAATTGAAAG-3’
	Antisense	5’-TGCATCAACGCACCCAGTC-3’
PPOX	Sense	5’-GCCCTTGAAACCCACCTGACTA-3’
	Antisense	5’-ACTAATAACGTGGTCAGCCTCCAGA-3’
FECH	Sense	5’-AGGCCATTAAGATGGATGTTGGAA-3’
	Antisense	5’-CTGTCAGAGTGAAGGCTCACAAGAA-3’
PEPT1	Sense	5’-TCACCTGTGGCGAAGTGGTC-3’
	Antisense	5’-GCCACGATGAGCACAATGATG-3’
ABCG2	Sense	5’-GCAACCATCAATTCAGGTCAAGA-3’
	Antisense	5’-GAAACACAACACTTGGCTGTAGCA-3’
Actin	Sense	5’-TGGCACCCAGCACAATGAA-3’
	Antisense	5’-CTAAGTCATAGTCCGCCTAGAAGCA-3’

The amplification conditions included 30 s at 95°C; 45 cycles at 95°C for 5 s and 60°C for 60 s each; dissociation for 15 s at 95°C and 30 s at 60°C; and then 15 s at 95°C on a Thermal Cycler Dice Real-Time System.

Thermal Cycler Dice Real-Time System analysis software (TaKaRa, Shiga, Japan) was used for data analysis. The Ct values (cycle threshold) were calculated using the crossing-point method, and the relative target mRNA expression levels were measured by comparison with a standard curve. The results for each sample were normalized to *ACTB*, a housekeeping gene encoding beta actin.

### Analysis of Mitochondrial PpIX Productivity

Cells (2.8 × 10^7^ cells) were washed twice with PBS and collected with a scraper. Pellets were lysed with exctraction buffer (10 mM HEPES, 200 mM mannitol, 70 mM sucrose, 1 mM EGTA, pH 7.5) and homogenized with a glass homogenizer (Wheaton, Millville, New Jersey, USA). The homogenate was centrifuged at 600 ×*g* for 10 min to remove debris. The resulting supernatant were centrifuged at 11,000 ×*g* for 10 min and washed with extraction buffer. The pellet was resuspended in storage buffer (10 mM HEPES, 250 mM sucrose, 1 mM ATP, 0.08 mM ADP, 5 mM sodium succinate, 2 mM K_2_HPO_4_, 1 mM DTT, pH 7.4) and used as the mitochondrial fraction. Protein concentrations were determined using the Bradford assay (Bio-Rad Laboratories, Hercules, California, USA). To prepare CPgenIII-containing medium, cells (2 × 10^6^ cells) were seeded in a 6-cm dish and incubated for 24 h. Cells were then incubated with 4 mL medium containing 1 mM ALA for 24 h under an atmosphere containing 0.1% O_2_. The medium was collected and centrifuged at 1,500 ×*g* for 10 min. The supernatant was used as CPgenIII-containing medium. The mitochondrial fraction (10 μg) was diluted with 100 μL CPgenIII-containing medium. N-methyl-PpIX (1 μM) was added and the mixture was incubated under an atmosphere containing 21% O_2_ or 0.1% O_2_ for 12 h. Porphyrins were identified by HPLC as described above.

### Immunoblotting

Immunoblot analyses were carried out as previously described [15]. We used rabbit polyclonal anti-ALAD (Abcam, Cambridge, UK), mouse monoclonal anti-Actin (MP Biomedicals, Santa Ana, California, USA), mouse monoclonal anti-COX4I1 (Santa Cruz Biotechnology, Dallas, Texas, USA), goat polyclonal anti-HIF-1α (R&D systems, Minneapolis, USA), rabbit polyclonal anti-HSP90 (Santa Cruz Biotechnology, Dallas, Texas, USA), and mouse monoclonal anti-PPOX (Abcam, Cambridge, UK) as the primary antibodies, depending on the purpose of the immunoblot analysis. For the second antibody, we used horseradish peroxidase (HRP)-conjugated anti-mouse IgG antibody, HRP-conjugated anti-rabbit IgG antibody (Cell Signaling Technology, Beverly, Massachusetts, USA), and HRP-conjugated anti-goat IgG antibody (Santa Cruz Biotechnology, Dallas, Texas, USA) at 1:3000 dilution.

### ALA-PDT

Cells were incubated with ALA in a 6-well plate (1 × 10^5^ cells/well) under an atmosphere containing 5% CO_2_ at 37°C for 24 h. Cells were then exposed to LED irradiation for 5 min (630 nm, 1080 mJ/cm^2^) by placement of the plate below an LED irradiation unit (provided by SBI Pharma CO., LTD., Tokyo, Japan) as previously described [16]. Cells were further incubated in the dark under an atmosphere containing 5% CO_2_ at 37°C for 24 h. Cell viability was measured using the MTT assay, as previously described [16].

## Results

### Reduction of PpIX Synthesis and Promotion of CPIII Excretion Is Oxygen-dependent

**Fig 1:**
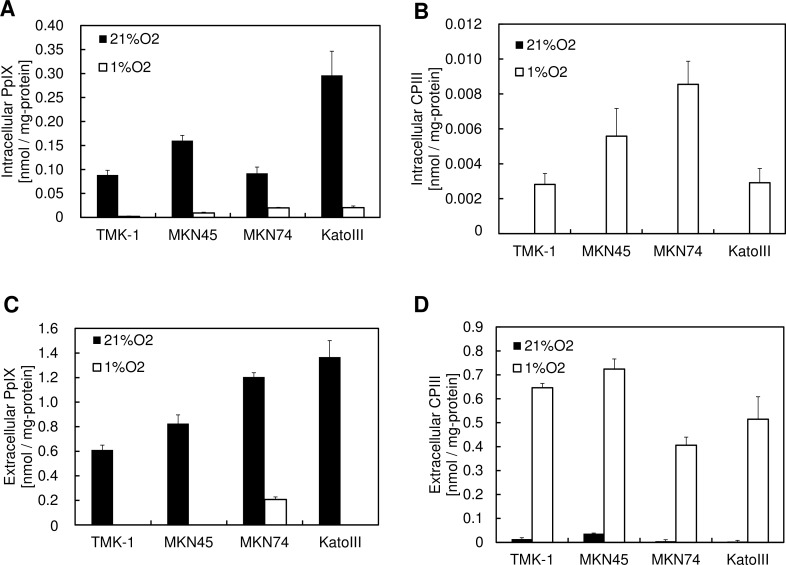
Generality of PpIX reduction and CPIII excretion after ALA administration under hypoxic conditions. (A-D) Each cell line was incubated with 1 mM ALA for 24 h under atmospheres containing 21% O_2_ or 1% O_2_. (A) Intracellular PpIX, (B) intracellular CPIII, (C) extracellular PpIX, and (D) extracellular CPIII were measured using HPLC. Data are expressed as the means ± S.D. from multi-replicated (n = 3) experiments.

**Fig 2:**
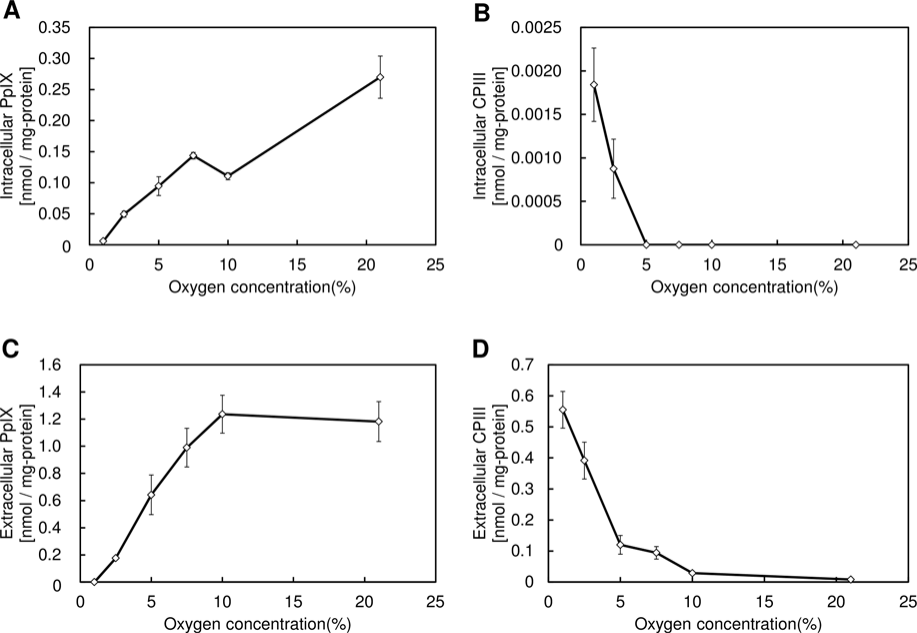
Oxygen dependence of PpIX reduction and CPIII excretion under hypoxic conditions. (A-D) KatoIII gastric cancer cells were incubated with 1 mM ALA for 24 h under atmospheres containing 21%, 10%, 7.5%, 5%, 2.5%, or 1% O_2_. (A) Intracellular PpIX, (B) intracellular CPIII, (C) extracellular PpIX, and (D) extracellular CPIII were measured using HPLC. Data are expressed as the means ± S.D. from multi-replicated (n = 3) experiments.

Our previous studies have shown that PpIX synthesis is decreased and CPIII excretion is increased during hypoxia [15]. To confirm the generality of this phenomenon, we assessed the accumulation of these porphyrins in 4 gastric cancer cell lines during hypoxia. All 4 cancer cell lines accumulated intracellular and extracellular PpIX during normoxia, whereas PpIX accumulation was decreased and CPIII excretion was increased during hypoxia (Fig 1). To examine the details of this alteration of porphyrin biosynthesis during hypoxia, we examined the accumulation of PpIX and CPIII after altering the oxygen concentration. Fig 2 shows that a gradual reduction in PpIX synthesis and a promotion of CPIII excretion were observed in atmospheres containing <7.5% O_2_, indicating that the alteration of porphyrin biosynthesis is caused by a decrease in the cellular oxygen concentration. Thus, these results indicate that the alteration of porphyrin biosynthesis is an oxygen concentration-dependent phenomenon.

### Transcriptional regulation is not associated with the alteration of porphyrin biosynthesis during hypoxia

**Fig 3:**
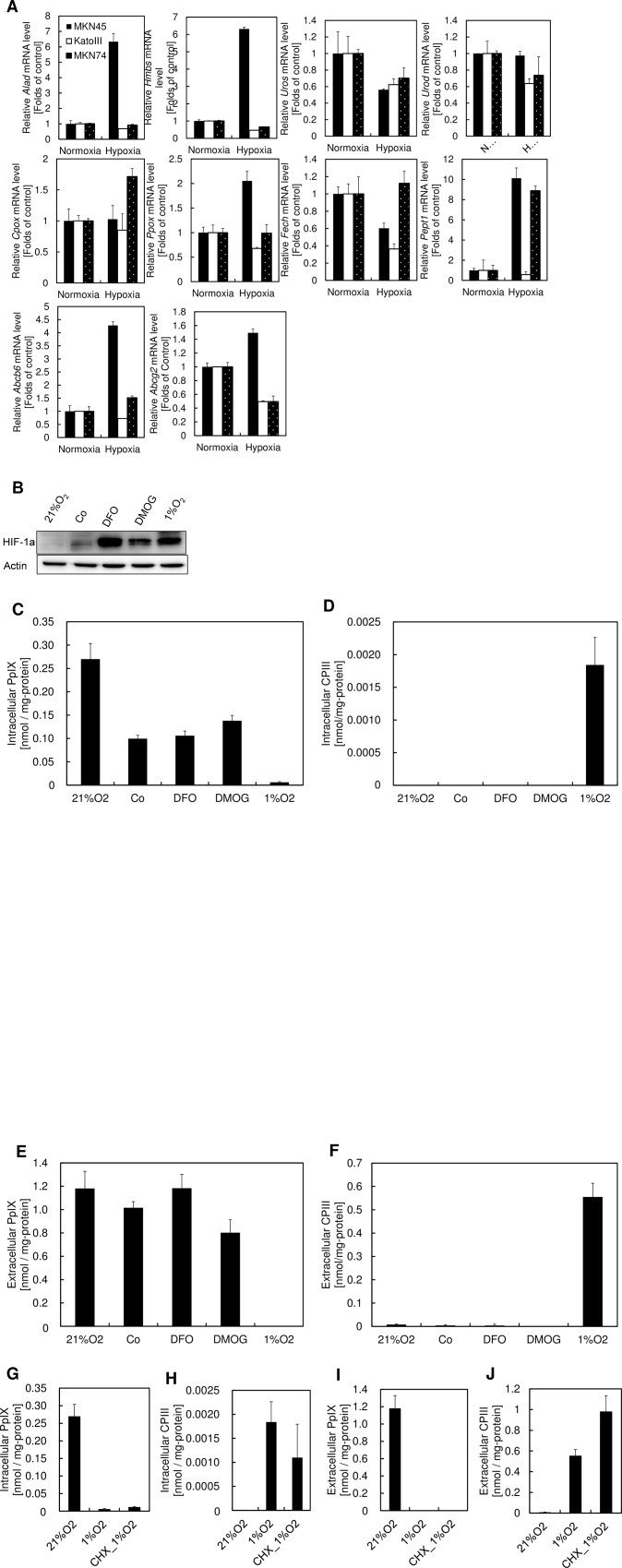
Transcriptional regulation is not associated with the alteration of porphyrin biosynthesis during hypoxia. (A) MKN45, KatoIII, and MKN74 cells were incubated for 24 h under atmospheres containing 21% O_2_ or 1% O_2_. mRNA expression levels of *ALAD*, *HMBS*, *UROS*, *UROD*, *CPOX*, *PPOX*, *FECH*, *PEPT1*, *ABCB6*, and *ABCG2* in each cell line were analyzed by quantitative PCR. Data are expressed as the means ± S.D. from multi-replicated (n = 2) experiments. *ALAD*, aminolevulinic acid dehydrogenase; *HMBS*, hydroxymethylbilane synthase; *UROS*, uroporphyrinogen III synthase; *UROD*, uroporphyrinogen III decarboxylase; *CPOX*, coproporphyrinogen III oxidase; *PPOX*, protoporphyrinogen oxidase; *FECH*, ferrochelatase; *PEPT1*, Peptide transporter 1 (B) KatoIII cells were incubated with 100 μM CoCl_2_, 100 μM deferoxamine (DFO), or 1 mM dimethyloxaloglycine (DMOG) for 24 h under atmospheres containing 21% O_2_ or 1% O_2_. Immunoblots were carried out using antibodies against HIF-1α and actin. (C-F) KatoIII cells were incubated with 1 mM ALA and 100 μM CoCl_2_, 100 μM DFO, or 1 mM DMOG for 24 h under atmospheres containing 21% O_2_ or 1% O_2_. (C) Intracellular PpIX, (D) intracellular CPIII, (E) extracellular PpIX and (F) extracellular CPIII were measured using HPLC. (G-J) KatoIII cells were incubated with 1 mM ALA and 10 μg/mL cyclohexamide. (G) Intracellular PpIX, (H) intracellular CPIII, (I) extracellular PpIX and (J) extracellular CPIII were measured using HPLC. Data are expressed as the means ± S.D. from multi-replicated (n = 3) experiments.

To examine whether the alteration of porphyrin biosynthesis during hypoxia is caused by transcriptional regulation of porphyrin biosynthetic enzymes or transporters, we compared expression of the genes encoding porphyrin biosynthetic enzymes and transporters during normoxia and hypoxia. Except for the gene encoding uroporphyrinogen III synthase, concordant alteration of gene expression was not observed for genes involved in porphyrin-heme biosynthesis pathway during hypoxia (Fig 3A). Because uroporphyrinogen III synthase isn’t the rate-limiting enzyme of porphyrin biosynthesis, this result indicates that the expression levels of porphyrin biosynthesis genes are not associated with the alteration of porphyrin biosynthesis during hypoxia (Fig 3A).

HIF-1α plays a central role in the adaptive response in hypoxia [9]. To examine the effect of HIF-1α on the alteration of porphyrin biosynthesis during hypoxia, PpIX and CPIII production levels were measured in the presence of HIF-1α inducers. CoCl_2_, a competitive inhibitor, and deferoxamine (DFO), a chelator of Fe ion, inhibit the activity of prolyl hydroxylases (PHDs) and stabilize HIF-1α protein [17,18]. Dimethyloxalylglycine, an analog of 2-oxoglutarate, also inhibits the activity of PHDs [19]. Using these inhibitors, the HIF-1α expression level was increased during normoxia (Fig 3B). However, PpIX and CPIII levels were almost unchanged despite HIF-1α activation (Fig 3C–3F). These results indicate that HIF-1α is not associated with the alteration of porphyrin biosynthesis during hypoxia.

Next, we examined the effect of overall genetic responses to hypoxia on PpIX and CPIII production. Cycloheximide, an inhibitor of protein synthesis, had almost no effect on the alteration of porphyrin biosynthesis during hypoxia (Fig 3G–3J), indicating that the overall genetic response of hypoxia is not associated with porphyrin biosynthesis. Altogether, these results suggest that transcriptional regulation during hypoxia is not a main factor in the alteration of porphyrin biosynthesis during hypoxia.

### Decreased Activity of Mitochondrial Porphyrin Biosynthetic Enzymes Leads to Reduction of PpIX Synthesis

**Fig 4:**
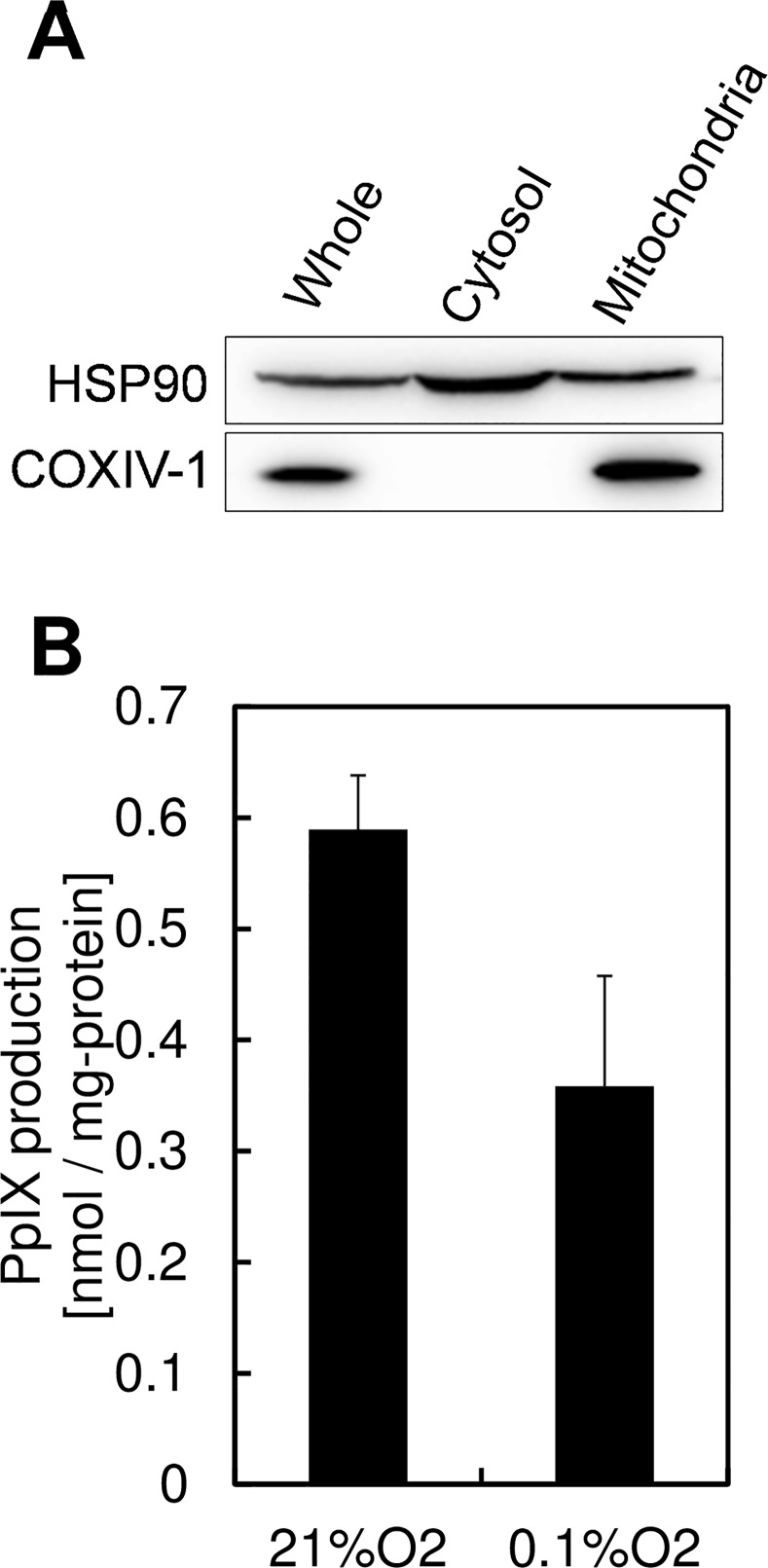
Mitochondrial PpIX production was reduced under hypoxic conditions. (A) KatoIII mitochondria were isolated using extraction buffer. Immunoblots were carried out using antibodies against HSP90 and COX IV-1. (B) Mitochondrial PpIX production was measured using isolated mitochondria and CPgenIII-containing medium. CPgenIII-containing medium was prepared by incubating KatoIII cells with 1 mM ALA for 24 h under an atmosphere containing 0.1% O2. CPgenIII-containing medium were added to isolated mitochondria and the mixtures were incubated for 12 h under atmospheres containing 21% O2 or 0.1% O2. PpIX was measured using HPLC. Data are expressed as the means ± S.D. from multi-replicated (n = 3) experiments.

Because production of PpIX and CPIII was changed in an oxygen concentration-dependent manner, we focused on the oxygen-dependent enzymes in porphyrin biosynthesis. Because coproporphyrinogen oxidase and protoporphyrinogen oxidase, the final 2 enzymes in PpIX synthesis, require oxygen molecules in their enzymatic activity [20], we hypothesized that the activities of coproporphyrinogen III oxidase and protoporphyrinogen oxidase are diminished by a lack of oxygen, resulting in a decrease in PpIX production during hypoxia. To test this hypothesis, the ability of mitochondria to produce PpIX was measured by comparing the normoxic and hypoxic cultivation of extracted mitochondria in the presence of CPgenIII-containing medium. Mitochondria was isolated by centrifugation method. Mitochondrial condensation was confirmed by expression of cytochrome oxidase IV isoform 1, the mitochondria specific enzyme. (Fig 4A), and CPgenIII-containing medium was prepared by incubating KatoIII cells with 1 mM ALA under hypoxic conditions. Fig 4B shows that hypoxic cultivation of mitochondria with CPgenIII-containing medium decreased PpIX production, which indicates that oxygen limitation in mitochondria results in a decrease in PpIX synthesis. This effect may result from the oxygen restriction on porphyrin biosynthetic enzymes.

### Inhibition of Mitochondrial Respiration Complexes Recovers the Production of PpIX in Hypoxia

**Fig 5:**
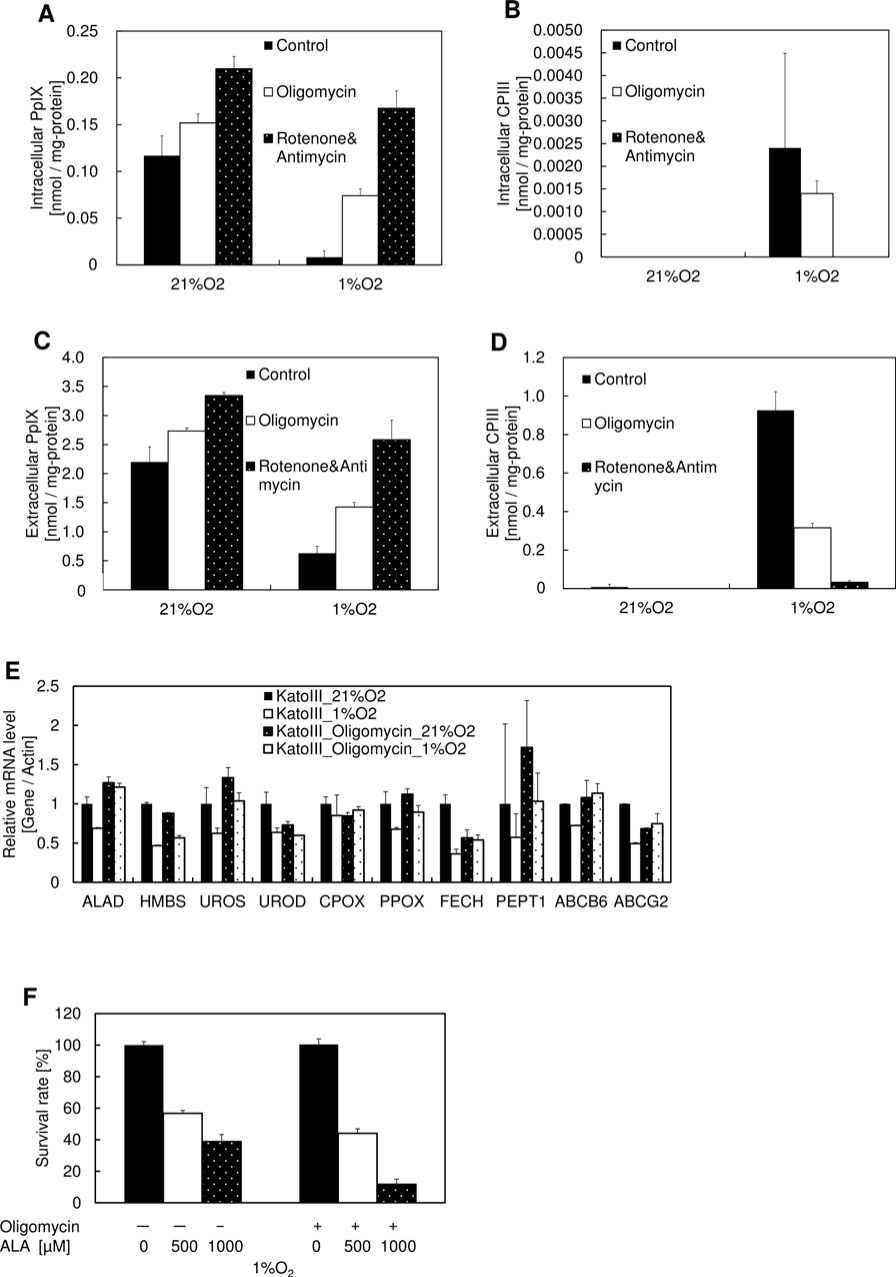
Effect of respiration inhibitors on PpIX production and photodynamic therapy. (A-D) KatoIII cells were incubated with 1 mM ALA and 0.1 μM oligomycin, 1 μM rotenone, or 1 μM antimycin for 24 h under atmospheres containing 21% O_2_ or 1% O_2_. (A) Intracellular PpIX, (B) intracellular CPIII, (C) extracellular PpIX, and (D) extracellular CPIII were measured using HPLC. Data are expressed as the means ± S.D. from multi-replicated (n = 3) experiments. (E) KatoIII cells were incubated with 0.1 μM oligomycin for 24 h under atmospheres containing 21% O_2_ or 1% O_2_. mRNA expression levels of *ALAD*, *HMBS*, *UROS*, *UROD*, *CPOX*, *PPOX*, *FECH*, *PEPT1*, *ABCB6*, and *ABCG2* were analyzed by quantitative PCR. Data are expressed as the means ± S.D. from multi-replicated (n = 2) experiments. *ALAD*, aminolevulinic acid dehydrogenase; *HMBS*, hydroxymethylbilane synthase; *UROS*, uroporphyrinogen III synthase; *UROD*, uroporphyrinogen III decarboxylase; *CPOX*, coproporphyrinogen III oxidase; *PPOX*, protoporphyrinogen oxidase; *FECH*, ferrochelatase; *PEPT1*, Peptide transporter 1 (F) KatoIII cells were incubated with 1 mM ALA and 0.1 μM Oligomycin for 24 h under atmospheres containing 21% O_2_ or 1% O_2_. Exposure to light was performed under an atmosphere containing 21% O_2_ and cell viability was measured using the MTT assay. Data are expressed as the means ± S.D. from multi-replicated (n = 3) experiments.

Mitochondrial respiration is the main consumer of cellular oxygen [21]. To estimate the effect of respiration on PpIX production, we compared PpIX production and CPIII excretion in the presence of respiration complex inhibitors. Fig 5A–5D shows that treatment using the Complex I inhibitor rotenone, the Complex III inhibitor antimycin, or the Complex V inhibitor oligomycin recovered PpIX production during hypoxia. Although ABCB6 was reported to import CPgenIII in an ATP-dependent manner [22,23], inhibition of Complex I, III or V, which reduces cellular ATP, didn’t decrease the production of PpIX (Fig 5A–5D). Thus, ABCB6 may not be associated with the alteration of porphyrin biosynthesis during hypoxia. The expression levels of porphyrin biosynthetic enzymes and transporters were almost unchanged (Fig 5E), indicating no association between transcriptional regulation and the alteration of porphyrin biosynthesis during hypoxia, similar to Fig 3A. In addition, the combination of ALA and oligomycin enhanced the efficacy of ALA-PDT in hypoxic cancer cells (Fig 5F). These results collectively indicate that the preferential consumption of mitochondrial oxygen by respiration limits the activity of coproporphyrinogen III oxidase and protoporphyrinogen oxidase, resulting in a reduction in PpIX synthesis during hypoxia, which enhances the effect of ALA-PDT and PDD during hypoxia.

## Discussion

The phenomenon of PpIX accumulation in cancer cells after administration of ALA is widely used for the photodiagnosis of tumors in the clinic [1–3]. Whereas numerous studies have focused on the mechanism of PpIX accumulation in cancer cells, few studies have focused on the mechanism of CPIII accumulation. CPIII has been found in the blood and urine samples of cancer patients and can be useful for the diagnosis of cancer [24]. In this report, we demonstrated that mitochondrial oxygen depletion reduces PpIX and causes the excretion of CPIII during hypoxia, which was not associated with gene expression. We proposed a model in which the enzymes of mitochondrial respiration and porphyrin biosynthesis compete for molecular oxygen during hypoxia, resulting in reduced PpIX production.

According to previous studies, the intracellular CPgenIII level during hypoxia is over 100 times lower than the extracellular CPgenIII level. Also, the intracellular CPgenIII level during hypoxia is far lower than the intracellular PpIX level during normoxia. These results lead to the hypothesis that hypoxia promotes a certain mechanism that excretes CPgenIII to decrease the intracellular CPgenIII level. However, respiration complex inhibitors increased the production of PpIX and reduced the production of CPgenIII during hypoxia (Fig 5A–5D). These results lead to the hypothesis that the main factor responsible for CPgenIII production during hypoxia is not the activation of transporters, but the reduced activities of porphyrin biosynthetic enzymes. The ratio of intracellular to extracellular CPIII was not changed, even in the presence of respiration complex inhibitors. Thus, the reduction of CPgenIII and the promotion of CPgenIII excretion may work via different mechanisms. ABCB6 is the intracellular CPgenIII transporter requiring ATP in its activity. Inhibitors of respiration complexes inhibits cellular ATP generation, which could inhibit CPgenIII transport by inhibition of ABCB6 activity via ATP inhibition. However, inhibitors of respiration complexes didn’t affect the accumulation of PpIX even in 21% O_2_ (Fig 5A–5D), indicating ABCB6 is not the rate-limiting enzyme in ALA treated cells.

Interestingly, although HIF-1, which is induced by hypoxia, represses the activity of respiration complexes and COX [25], the inhibition of respiration complexes recovers the production of PpIX during hypoxia. This result indicates that the inhibition of respiration complexes by HIF-1 is incomplete, and that respiration complexes use the maximum capacity of oxygen in the mitochondria: around 1% O_2_. This result also indicates that respiration complexes and porphyrin biosynthetic enzymes in mitochondria compete with each other during hypoxia. However, it is unclear whether the physiological level of porphyrin biosynthesis is restricted by respiration complexes during hypoxia. Further studies of the availability of molecular oxygen in the mitochondria for enzymes other than the respiration complexes during hypoxia are required.

Fig 5F shows that increasing oxygen availability for porphyrin biosynthetic enzymes can enhance the effect of ALA-PDT during hypoxia. Physiological oxygen concentration of mammalian tissues is around 3 to 6%, which is abundant than tumor tissues [26]. In addition, Fig 2 shows that CPIII accumulation in 3 to 6% O_2_ is much lower than that in 1% O_2_. Thus, the inhibition of respiration complexes would have little effect on PpIX production in normal cells. On the other hand, inhibition of respiration complexes increased PpIX production in hypoxic cancer cells. Altogether, combination of ALA and respiration complexes inhibitors might enhance the specificity of ALA-PDT and PDD.

In conclusion, we discovered a relationship between mitochondrial respiration complexes and porphyrin biosynthetic enzymes that affects PpIX production during hypoxia. Targeting respiration complexes is, therefore, a compelling rationale for enhancing the efficacy of ALA-mediated treatment and diagnosis.

## References

[1] Stummer W, Pichlmeier U, Meinel T, Wiestler OD, Zanella F, Reulen HJ. (2006) Fluorescence-guided surgery with 5-aminolevulinic acid for resection of malignant glioma: a randomised controlled multicentre phase III trial. Lancet Oncol 7: 392–401. 16648043 10.1016/S1470-2045(06)70665-9

[2] Inoue K, Fukuhara H, Shimamoto T, Kamada M, Iiyama T, Miyamura M, et al (2011) Comparison between intravesical and oral administration of 5-aminolevulinic acid in the clinical benefit of photodynamic diagnosis for non-muscle invasive bladder cancer. Cancer 118: 1062–1074. 10.1002/cncr.26378 21773973

[3] Fukuhara H, Inoue K, Satake H, Tamura K, Karashima T, Yamasaki I, et al (2011) Preliminary experience of photodynamic diagnosis of positive margin during radical prostatectomy by oral 5-aminolevulinic acid. Int J Urol 18: 585–591. 10.1111/j.1442-2042.2011.02789.x 21658132

[4] Wilson WR, Hay MP. (2011) Targeting hypoxia in cancer therapy. Nature Reviews Cancer 11: 393–410. 10.1038/nrc3064 21606941

[5] Gray LH, Conger AD, Ebert M, Hornsey S, Scott OC. (1953) The concentration of oxygen dissolved in tissues at the time of irradiation as a factor in radiotherapy. Br. J. Radiol. 26: 638–648. 13106296 10.1259/0007-1285-26-312-638

[6] Brown JM, Wilson WR. (2004) Exploiting tumour hypoxia in cancer treatment. Nat. Rev. Cancer 4: 437–447. 15170446 10.1038/nrc1367

[7] Georgakoudi I, Keng PC, Foster TH. (1999) Hypoxia significantly reduces aminolaevulinic acid-induced protoporphyrin IX synthesis in EMT6 cells. British journal of cancer 79: 1372–1377 10188878 10.1038/sj.bjc.6690220 PMC2362734

[8] Wyld L, Reed MW, Brown NJ. (1998) The influence of hypoxia and pH on aminolaevulinic acid-induced photodynamic therapy in bladder cancer cells in vitro. British journal of cancer 77: 1621–1627. 9635837 10.1038/bjc.1998.265 PMC2150064

[9] Semenza GL. (2003) Targeting HIF-1 for cancer therapy. Nature reviews cancer 3: 721–732. 13130303 10.1038/nrc1187

[10] Chepelev NL, Willmore WG. (2011) Regulation of iron pathways in response to hypoxia. Free Radic Biol Med. 50: 645–666. 10.1016/j.freeradbiomed.2010.12.023 21185934

[11] Tomaro ML, Frydman RB, Gutnisky A, Sburlati A. (1981) Induction of porphobilinogen oxygenase and porphobilinogen deaminase in rat blood under conditions of erythropoietic stress. Biochimica et Biophysica Acta 676: 31–42. 7260110 10.1016/0304-4165(81)90006-4

[12] Liu YL, Ang SO, Weigent DA, Prchal JT, Bloomer JR. (2004) Regulation of ferrochelatase gene expression by hypoxia. Life Sci. 75: 2035–2043. 15312748 10.1016/j.lfs.2004.03.027

[13] Vargas PD, Furuyama K, Sassa S, Shibahara S. (2008) Hypoxia decreases the expression of the two enzymes responsible for producing linear and cyclic tetrapyrroles in the heme biosynthetic pathway. FEBS J. 275: 5947–5959. 10.1111/j.1742-4658.2008.06723.x 19021769

[14] Krishnamurthy P, Ross DD, Nakanishi T, Bailey-Dell K, Zhou S, Mercer KE, et al (2004) The stem cell marker Bcrp/ABCG2 enhances hypoxic cell survival through interactions with heme. J Biol Chem. 279: 24218–24225. 15044468 10.1074/jbc.M313599200

[15] Matsumoto K, Hagiya Y, Endo Y, Nakajima M, Ishizuka M, Tanaka T, et al (2015) Effects of plasma membrane ABCB6 on 5-aminolevulinic acid (ALA)-induced porphyrin accumulation in vitro: Tumor cell response to hypoxia. Photodiagnosis Photodyn Ther. 12: 45–51. 10.1016/j.pdpdt.2014.12.008 25573285

[16] Hagiya Y, Endo Y, Yonemura Y, Takahashi K, Ishizuka M, Abe F, et al (2012) Pivotal roles of peptide transporter PEPT1 and ATP-binding cassette (ABC) transporter ABCG2 in 5-aminolevulinic acid (ALA)-based photocytotoxicity of gastric cancer cells in vitro. Photodiagnosis Photodyn Ther. 9:204–214. 10.1016/j.pdpdt.2011.12.004 22959800

[17] Wang GL, Semenza GL. (1993) Desferrioxamine induces erythropoietin gene expression and hypoxia-inducible factor 1 DNA-binding activity: implications for models of hypoxia signal transduction. Blood 82: 3610–3615. 8260699

[18] Yuan Y, Hilliard G, Ferguson T, Millhorn DE. (2003) Cobalt inhibits the interaction between hypoxia-inducible factor-α and von Hippel-Lindau protein by direct binding to hypoxia-inducible factor-α. J Biol Chem. 278: 15911–15916. 12606543 10.1074/jbc.M300463200

[19] Jaakkola P, Mole DR, Tian YM, Wilson MI, Gielbert J, Gaskell SJ, et al (2001) Targeting of HIF-α to the von Hippel-Lindau ubiquitylation complex by O2-regulated prolyl hydroxylation. Science 292: 468–472. 11292861 10.1126/science.1059796

[20] Sano S, Granick S. (1961) Mitochondrial coproporphyrinogen oxidase and protoporphyrin formation. J Biol Chem. 236: 1173–1180. 13746277

[21] Wilson DF, Rumsey WL, Green TJ, Vanderkooi JM. (1988) The oxygen dependence of mitochondrial oxidative phosphorylation measured by a new optical method for measuring oxygen concentration. J Biol Chem. 263: 2712–2718. 2830260

[22] Krishnamurthy PC, Du G, Fukuda Y, Sun D, Sampath J, Mercer KE, et al (2006) Identification of a mammalian mitochondrial porphyrin transporter. Nature 443: 586–589. 17006453 10.1038/nature05125

[23] Rebeiz N, Arkins S, Kelley KW, Rebeiz CA. (1996) Enhancement of coproporphyrinogen III transport into isolated transformed leukocyte mitochondria by ATP. Arch Biochem Biophys. 333: 475–481. 8809089 10.1006/abbi.1996.0417

[24] Inoue K, Ota U, Ishizuka M, Kawada C, Fukuhara H, Shuin T, et al (2013) Porphyrins as urinary biomarkers for bladder cancer after 5-aminolevulinic acid (ALA) administration: The potential of photodynamic screening for tumors. Photodiagnosis Photodyn Ther. 10: 484–489. 10.1016/j.pdpdt.2013.05.002 24284101

[25] Papandreou I, Cairns RA, Fontana L, Lim AL, Denko NC. (2006) HIF-1 mediates adaptation to hypoxia by actively downregulating mitochondrial oxygen consumption. Cell metabolism 3: 187–197. 16517406 10.1016/j.cmet.2006.01.012

[26] Meyron-Holtz EG, Ghosh MC, Rouault TA. (2004) Mammalian tissue oxygen levels modulate iron-regulatory protein activities in vivo. Science 306: 2087–90. 15604406 10.1126/science.1103786

